# Structural prediction of RNA switches using conditional base-pair probabilities

**DOI:** 10.1371/journal.pone.0217625

**Published:** 2019-06-12

**Authors:** Amirhossein Manzourolajdad, John L. Spouge

**Affiliations:** National Center for Biotechnology Information, National Library of Medicine, National Institutes of Health, Bethesda, Maryland, United States of America; Weizmann Institute of Science, ISRAEL

## Abstract

An RNA switch triggers biological functions by toggling between two conformations. RNA switches include bacterial riboswitches, where ligand binding can stabilize a bound structure. For RNAs with only one stable structure, structural prediction usually just requires a straightforward free energy minimization, but for an RNA switch, the prediction of a less stable alternative structure is often computationally costly and even problematic. The current sampling-clustering method predicts stable and alternative structures by partitioning structures sampled from the energy landscape into two clusters, but it is very time-consuming. Instead, we predict the alternative structure of an RNA switch from conditional probability calculations within the energy landscape. First, our method excludes base pairs related to the most stable structure in the energy landscape. Then, it detects stable stems (“seeds”) in the remaining landscape. Finally, it folds an alternative structure prediction around a seed. While having comparable riboswitch classification performance, the conditional-probability computations had fewer adjustable parameters, offered greater predictive flexibility, and were more than one thousand times faster than the sampling step alone in sampling-clustering predictions, the competing standard. Overall, the described approach helps traverse thermodynamically improbable energy landscapes to find biologically significant substructures and structures rapidly and effectively.

## Introduction

In many organisms, structural rearrangements of RNA switches trigger biological functions. In bacteria, RNA switches regulate gene expression of mRNA downstream from them [1, 2]. In eukaryotes, they regulate alternative splicing [3]. In viruses, they can be critical in various stages of the viral life cycle, regulating rates of replication, transcription, RNA dimerization, etc. [4–9]. Plasticity, the ability to assume more than one structure, can also enhance the adaptability of RNA [10] by permitting it to accommodate distinct conformational phenotypes with only small perturbations to its genotype.

Current scientific strategies have produced an abundance of prokaryotic data, so most known RNA switches are in bacteria. To find putative riboswitches in prokaryotic sequences, common approaches rely on the assumption that the biologic mechanism of the riboswitch is at least partially conserved across certain bacteria. Hence, they locate conserved structural elements upstream of homologous coding regions [11, 12]. The Rfam database [13] provides a publicly available set of regulatory RNAs, including riboswitches. Although known riboswitch families are already structurally and functionally diverse [14–16] they probably represent only a small fraction of all bacterial riboswitches [17]. In any case, they provide invaluable examples of RNA molecules with stable alternative conformations.

**Fig 1:**
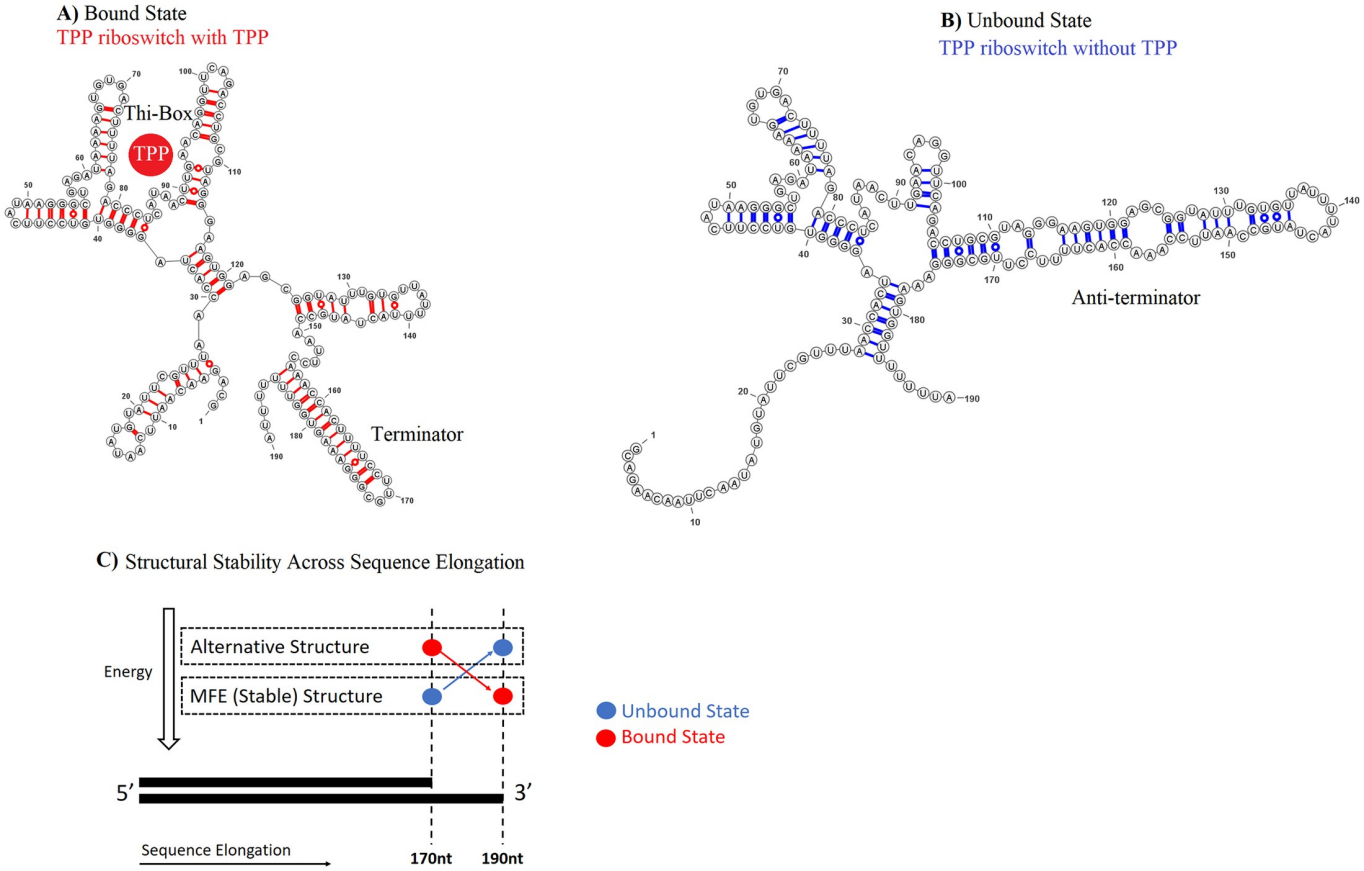
Two structural states of the thiamine pyrophosphate (TPP) riboswitch in *Bacillus subtilis*. [18]. A) Riboswitch when TPP is present. TPP ligand approximate location shown as solid circle. Thi-Box and terminator are formed. B) Riboswitch when TPP is absent. Anti-terminator is formed. The aptamer region is roughly between 1nt-120nt and the expression platform is roughly between 120nt-190nt (A and B). C) Folding energy of the TPP riboswitch. The unbound state is more stable, when the elongation is 170nt; the bound state, when the elongation increases to 190nt.

After selective binding to a specific ligand, metabolite, or uncharged transfer RNA, the structure of a bacterial riboswitch toggles from unbound to the bound state. The conformational change then influences the expression of a proximal downstream gene. Most riboswitches contain two substructures: (1) an aptamer, which binds the ligand or metabolite, and which is usually structurally conserved; and (2) an expression platform, which undergoes allosteric rearrangement to regulate the gene. The thiamine pyrophosphate (TPP) riboswitch is a typical case illustrating regulation through a structural rearrangement. Fig 1 illustrates schematics of the bound and unbound conformations of the TPP riboswitch in *Bacillus subtilis* [18]. Fig 1A illustrates the bound state, where the aptamer in the RNA substructure surrounds TPP. In the bound state, the aptamer (sequence coordinates 0nt-120nt) stabilizes, causing the downstream expression platform (coordinates 120nt-190nt) to form a terminator stem loop (Fig 1A). In the unbound state without the TPP ligand, however, the anti-terminator substructure in the expression platform is stable, disrupting the terminator substructure (Fig 1B). The structure of the riboswitch *cis*-regulates downstream genes by influencing their expression.

In typical riboswitches, the unbound structure has a lower computed folding energy than the bound structure, because the ligand is required to stabilize the bound state. Furthermore, folding kinetics sometimes fine-tune binding. Typically (with some exceptions, e.g., [19, 20]), the bound state becomes energetically more favorable as the mRNA transcribes and elongates, so in the presence of an adequate ligand concentration the structure increasingly switches to the bound state. For instance, for the TPP riboswitch at a sequence elongation of 170nt, the minimum free energy (MFE) structure [21–23] corresponds to the unbound state; but at an elongation of 190nt, the MFE structure corresponds to the bound state (Fig 1C). Therefore, the relative stability of the bound and unbound states of some riboswitches can vary under different sequence elongations or segment selections. For simplicity and consistency, we always refer to the less stable structure in the computed energy landscape, whether bound or unbound, as *the alternative* structure.

Prediction of alternative structures generally requires extensive analysis of the secondary structure energy landscape. There are many strategies for alternative structure prediction (reviewed in [24, 25]). Structural entropy of the RNA folding landscape [26, 27], graph-based representations [28], Markov-state models [29], abstract shapes [30–34], energy-band-based sampling [35] base-pair-distance-based alternative structure prediction [36, 37], and the more recent probability-corrected sampling [38] are just a few examples of using various modeling techniques to explore the RNA folding landscape.

The Boltzmann ensemble thermodynamic model is the basis of most predictive strategies. The model conceptualizes the RNA structure of the sequence under scrutiny as a Boltzmann ensemble, estimating ensemble energies from experimentally derived nearest-neighbor free energy parameters [39–43]. In the Boltzmann ensemble, the probability Pr(*S*) of each structure *S* is proportional to exp[−*E*(*S*)/*RT*], where *E*(*S*) is the free energy of *S*; *R*, the universal gas constant; and *T*, the folding temperature. Prediction of the alternative structure requires an efficient exploration of suboptimal (i.e., lower probability) structural configurations.

The Sampling-Clustering (SC) method is an important strategy for finding alternative structures [18, 44, 45]. First, suboptimal structures are sampled from the energy landscape, possibly under various temperatures to increase structural diversity. Then, the samples are partitioned into two clusters. The cluster containing the MFE structure corresponds to the stable structure while the other cluster corresponds to the alternative structure of the RNA under investigation. Although SC predicts some alternative structures accurately, it fails badly for others. Indeed, Boltzmann probabilities decrease exponentially with the free energy, with the potential to increase the SC computational runtimes exponentially [46]. For most riboswitches, the theoretical computational complexity poses no practical problem, however, because (1) the candidate riboswitch has only moderate length, or (2) exhaustive sampling is not required. For other riboswitches, however, the energy difference between the stable and the alternative structures (as much as 20 kcal/mol [37]) can severely slow the sampling. Furthermore, as the length *n* of the RNA sequence increases, the number of structures in the Boltzmann ensemble explodes exponentially, so traversing the ensemble explicitly becomes infeasible [47].

Instead of the time-consuming method of explicitly traversing structures of the ensemble, as SC does, in this work we infer the alternative structure from base-pair probabilities. Our Conditional-Probability (CP) method is as follows: First, the MFE structure is determined. Then, base-pair probabilities, conditioned on the absence of bonds between the MFE base pairs and their neighbors are calculated. McCaskill’s algorithm [48–50] efficiently calculates the exact base-pair probabilities. We then select a stem whose base pairs have high conditional probabilities as a “seed”. Finally, we determine the alternative structure by computing the structure with the lowest free energy of all structures containing the seed. UNAFold and Mfold [21, 50–53] implement the exact calculations, and in particular we use UNAFold (version 4.0.0) to calculate base-pair probabilities conditioned on the inclusion or exclusion of a given set of base pairs. Using an independent benchmark [54] consisting of riboswitches [18, 20, 24, 44, 55–71] (S1 Table) and the purine riboswitch family taken from Rfam [13], the Results section compares CP and SC predictions of alternative structures for both the prediction accuracy and computational times. Although CP prediction has very few adjustable parameters, the Results section also briefly describes the parameter space anecdotally in a single riboswitch to display the parameters’ impact on CP alternative structure prediction. The Discussion then examines some implications of our study.

## Materials and methods

**Sampling-Clustering (SC) procedure for predicting the alternative structure.** Given an RNA sequence, the energy landscape of the RNA is sampled at different temperatures starting from 300 structures at 37°C and 150 structures at each temperature value at six decile intervals towards the melting temperature of the RNA strand, totaling to 1200 samples per RNA. Sample numbers were selected according to same SC procedure used in [54] for comparison purposes. The samples are then partitioned into two clusters using k-means clustering, using base-pair Hamming distance *d*_*H*_ (the distance *d*_*H*_{*S'*,*S"*} counts the base pairs that are in either of the structures *S'* and *S"* but not in both). Denote the most energetically stable secondary structure configuration of a riboswitch by *S*_1_; the alternative secondary structure, by *S*_2_. As in [18], the computed MFE structure (denoted by $S_{1}^{*}$) then was used to predict the most stable structure *S*_1_. The lowest-energy structure of the cluster not containing the MFE structure (denoted by ${\hat{S}}_{2}^{*}$, the over-hat often denoting a sampled quantity in statistics) was used to predict the alternative structure *S*_2_.

**Barsacchi Dataset** consisting of 20 Riboswitches and 20 Non-coding (Non-riboswitch) RNAs. An extant benchmark, unchanged, provides sequences and structures for our evaluation of riboswitch classification [54]. Sequence lengths are identical to [54] for comparison purposes. We refer to the set of 20 riboswitches as 20-riboswitch set. The structures corresponding to twelve of those twenty riboswitches were provided by the authors and are used here for prediction accuracy calculations. We refer to this subset of sequences as the Barsacchi Structural dataset set (See S1 Table for names, lengths and number of actual structures available for each of the 20 riboswitches).

**The Purine Riboswitch dataset** consisted of the purine riboswitch family. First, the structurally conserved aptamers of purine riboswitch family RF00167 were downloaded from Rfam (133 sequences roughly 100-nt long), along with the consensus secondary structure which represented the bound state of the aptamer. Then, each aptamer sequence was extended 100nt downstream to capture the expression platform of the riboswitch as well. We mapped the consensus structure of the aptamer to the beginning of individual sequences.

**Structure visualization.** Structures in all figures were done with the aid of the VARNA software [72].

### Conditional-Probability prediction

We first computed the MFE secondary structure $S_{1}^{*}$ to predict *S*_1_. We then constructed an alternative structure $S_{2}^{*}$ to predict *S*_2_, as follows: Let [*i*·*j*] denote a base pair between sequence positions *i* and *j*. Given $S_{1}^{*}$, a base-pair-to-structure distance, and a dissimilarity threshold *τ*, we first build an excluded set $E^{\left(\tau \right)}$ consisting of base pairs close to base pairs $\left[i\cdot j\right]\in S_{1}^{*}$. Let us first describe base-pair-to-structure distance *δ*_bs_:

#### Base-pair distance

Our conditional probability predictions require a base-pair distance *δ*(*i*·*j*,*i'*·*j'*) between [*i*·*j*] and [*i'*·*j'*]. Base-pair distance *δ*_bb_ as defined in [22], is

$$\delta_{\text{bb}}\left(i\cdot j,i^{\prime }\cdot j^{\prime }\right)=\text{max}\left\{\left|i-i^{\prime }\right|,\left|j-j^{\prime }\right|\right\}.$$ (1)

Given a set of base pairs in a structure *S'*, [73] define the base-pair-to-structure distance

$$\delta_{\text{bs}}\left(i\cdot j,S^{\prime }\right)=\underset{i^{\prime }.j^{\prime }\in S^{\prime }}{\text{min}}\delta_{\text{bb}}\left(i\cdot j,i^{\prime }\cdot j^{\prime }\right).$$ (2)

Now, given a non-negative integer dissimilarity threshold *τ*, consider the excluded set $E^{\left(\tau \right)}$. All the base pairs in $E^{\left(\tau \right)}$ are selected such that their *δ*_bs_-distance to S* is less than or equal to *τ*:

$$E^{\left(\tau \right)}=\left\{\left[i\cdot j\right]:\delta_{\text{bs}}\left(i\cdot j,S_{1}^{*}\right)\leq \tau \right\},$$ (3)

#### McCaskill algorithm

Our methods make heavy use of the McCaskill algorithm [48] for calculating various probabilities related to the existence of a given base pair [*i*·*j*] in the structure of an RNA sequence at thermal equilibrium. Given the RNA sequence, the Boltzmann probability of its being in the structure *S* at thermal equilibrium is Pr(*S*) = *e*^−*E*(*S*)/*RT*^/*Z*, where the partition function *Z* = ∑*e*^−*E*(*S*)/*RT*^ is a normalizing factor. The McCaskill algorithm efficiently computes the exact probability *P*_*i*,*j*_ that *S* contains the base pair [*i*·*j*] through recursive calculations. The calculations combine various partition functions, given here in McCaskill’s notation. The restricted partition function $Q_{i,j}^{b}$ for bases from *i* to *j* inclusive is the sum of the Boltzmann weights *e*^−*E*(*S*)/*RT*^ over the structures *S*_*i*,*j*_ where the base pair [*i*·*j*] closes *S*_*i*,*j*_ into a loop structure. The full partition function $Q_{i,j}$*Q*_*i*,*j*_ for bases from *i* to *j* inclusive is the sum of *e*^−*E*(*S*)/*RT*^ over the corresponding structures *S*_*i*,*j*_ with and without the base pair [*i*·*j*], so for example the full partition function is *Z* = *Q*_1,*n*_. The probabilities *P*_*i*,*j*_ of individual base pairs [*i*·*j*] within the ensemble of structures *S* can be computed as *P*_*i*,*j*_ = ∑_*S*∋[*i*·*j*]_*e*^−*E*(*S*)/*RT*^/*Z* where the sum is over structures *S* containing [*i*·*j*]. McCaskill’s recursive calculations follow combinatorial patterns resembling algorithms for determining the MFE structure, computing first $Q_{i,j}^{b}$, then *Q*_*i*,*j*_, and finally *P*_*i*,*j*_ from previously computed values for smaller substructures. Due to complexity of McCaskill’s algorithm, we must refer the reader to [48] for details.

#### Conditional base-pair probabilities

Given a set of excluded base pairs E (e.g., $E^{\left(\tau \right)}$ above), the McCaskill algorithm can also compute the probability that a given base pair [*i*·*j*] exists, under the constraint that none of the base pairs in E are allowed. We denote the probability as

$$P_{i,j}^{\sim E}\equiv \text{Pr}\left\{\left[i\cdot j\right]\in S|\left[i^{\prime }\cdot j^{\prime }\right]\notin S,\;\forall \left[i^{\prime }\cdot j^{\prime }\right]\in E\right\}.$$ (4)

(In set theory, “~” often denotes complementation, so it helps suggest exclusion of E in the superscript of $P_{i,j}^{\sim E}$ in Eq (4)). Like calculating the unconstrained base-pair probabilities *P*_*i*,*j*_ above, the McCaskill algorithm can calculate the conditional base-pair probabilities in Eq (4) by using constrained partition functions. Our general plan is to constrain the alternative structure by avoiding all the base pairs in a neighborhood $E^{\left(\tau \right)}$ of the MFE structure, then to identify base pairs that the constrained structure is likely to contain, and then to fold the alternative structure around those base pairs.

The UNAfold (version 4.0.0) Software Package [50] calculates both the unconditional probabilities *P*_*i*,*j*_ and the conditional probabilities $P_{i,j}^{\sim E}$ efficiently with the McCaskill algorithm [48].

Given an excluded set E (e.g., $E^{\left(\tau \right)}$ above), next select a seed substructure L*, a longest stem whose every base pair *individually* has conditional probability higher than 0.5. Let *L* denote any stem (i.e., any ladder), where we permit *L* to contain bulges (i.e., single unpaired bases). Let #*L* count its base pairs.

$$L^{*}=\text{arg}{\text{max}}_{L}\left\{\#L|P_{i,j}^{\sim E}>0.5\;\text{for}\;\text{every}\;\left[i\cdot j\right]\in L\right\}$$

$$S_{2}^{*}=\text{arg}{\text{max}}_{S}\text{Pr}\left[S|\;L^{*}\in S\right]$$

Finally, $S_{2}^{*}$ is predicted as the lowest-energy structure that contains L*. Structure $S_{2}^{*}$ of the given RNA sequence serves to predict *S*_2_.

Because the seed lies outside E, by definition it always lies outside $S_{1}^{*}$, so $S_{2}^{*}\neq S_{1}^{*}$. Note that $S_{2}^{*}$ may still incidentally contain some originally excluded base pairs from E (or $S_{1}^{*}$, for that matter): the excluded base pairs in E are only used to compute the conditional probability distribution for selecting the seed. Once a seed is selected, it is the *only* constraint in energy minimization of $S_{2}^{*}$ (i.e., $S_{2}^{*}$ must contain it). Default calculations for seed-based predictions were performed at temperature 37°C and favored the version 3.0 energies of UNAfold, the most current energy parameters for RNA folding [40].

#### Generalization to other choices of seeds

We explored several criteria for seed selection. Empirically, the stem seed L* was most promising. Other seeds included the single base pair with highest $P_{i,j}^{\sim E}$ value and the union of all base pairs with $P_{i,j}^{\sim E}>0.5$.

#### Generalization to other temperatures

The most recent version of UNAfold (version 4.0.0) can make energy calculations at temperatures *T* = *T*_0_ other than 37°C, permitting the following generalized procedure. Predict structure $S_{1}^{*}$ at 37°C and construct the excluded set $E=E^{\left(\tau \right)}$, as above. Calculate all conditional base-pair probabilities $\left\{P_{i,j}^{\sim E}\right\}$ and stem seeds L* at various temperatures *T* = *T*_0_, to predict the alternative structure $S_{2}^{*T_{0}}$.

#### Generalization by iteration number

The procedure above could be considered the first step of an iterative process, where subsequent predictions $S_{i}^{*}$ (*i =* 3,4…) can be made by excluding the union $E=E_{i}=E_{i-1}\cup E_{i-2}$ of base pairs in previous predictions, and then identifying a seed $L_{i}^{*}$ from the resulting conditional probability distribution $\left\{P_{i,j}^{\sim E}\right\}$.

#### Notations

The alternative structure prediction $S_{2}^{*}$ is associated with adjustable parameters: threshold *τ = τ*_*0*_, temperature *T* = *T*_0_, iteration *i*, and the type of seed. It is denoted in full by *type-of-seed* $S_{i}^{*T_{0}}\left(\tau =\tau_{0}\right)$. For example, the alternative structure predicted with iteration 2 and using *τ* = 15, at temperature 70°C, and using a single base pair as the seed is denoted as *single-base-pair-seed* prediction $S_{2}^{*70{}^{\circ}\text{C}}\left(\tau =15\right)$. Our notation usually drops the default values, i.e. $S_{2}^{*}\equiv S_{2}^{*37{}^{\circ}\text{C}}\left(\tau =5\right)$ using stem seed L*. Unless otherwise stated, results used default parameters.

#### Normalized seed length

The number of base pairs within the seed was divided by the log of the sequence length SL, (#L*)/log_10_(SL), to derive Normalized Seed Length.

## Results

The Results section focuses on predicting the alternative structure. Here, the most energetically stable structure prediction is the MFE structure $S_{1}^{*}$, so its precise quality depends on the thermodynamic model. Furthermore, both the CP and SC methods use the MFE structure to predict the most stable structure. Therefore, the Results section omits comment on the stable structure prediction.

On the other hand, alternative structure prediction is sensitive to sequence elongation (i.e., the exact length chosen as the sequence under scrutiny). To avoid the possibility of cherry picking sequence lengths, our study took an independent “Barsacchi dataset” [54] (without change) as our dataset of 20 riboswitches and 20 non-riboswitches. The Barsacchi dataset provides a gold standard for RNA sequence and actual structures (if actual structures were available). The next subsection introduces the notations and concepts for the CP prediction using the TPP *tenA* riboswitch in *Bacillus subtilis* [18, 66] (*tenA* TPP) as a specific example.

### The CP prediction method applied to the *tenA* TPP riboswitch

The CP prediction method uses the computed MFE structure $S_{1}^{*}$ to predict the actual stable structure *S*_1_ (a standard prediction method, e.g., [54]); and prediction $S_{2}^{*}$ (as described in the Materials and methods section) to predict the actual alternative structure *S*_2_. The predicted alternative structure $S_{2}^{*}$ always has a higher computed energy than the predicted MFE structure $S_{1}^{*}$.

To predict $S_{2}^{*}$, we first determine a set of base pairs that are “close to” base pairs in $S_{1}^{*}$, namely all base pairs having distance less than or equal to dissimilarity threshold *τ*, $\delta_{\text{bs}}\left(i\cdot j,S_{1}^{*}\right)\leq \tau$ (see the Materials and methods section for precise mathematical definitions). Increasing *τ* therefore excludes progressively more base pairs from the alternative structure $S_{2}^{*}$. Our default dissimilarity measure is *τ* = 5. Let $E^{\left(\tau \right)}$ be the set of excluded base pairs. Conditioned on the absence of base pairs in $E^{\left(\tau \right)}$, the McCaskill algorithm yields the conditional probability of any other base pair. The Materials and Methods section defines a longest bulge-containing stem, L*, where every base pair has a conditional probability higher than 0.5, e.g., for *tenA* TPP, L* contained 7 base pairs. Finally, using the stem L* as a seed, constrained energy minimization yields $S_{2}^{*}$, which is the minimum energy structure containing L*.

**Fig 2:**
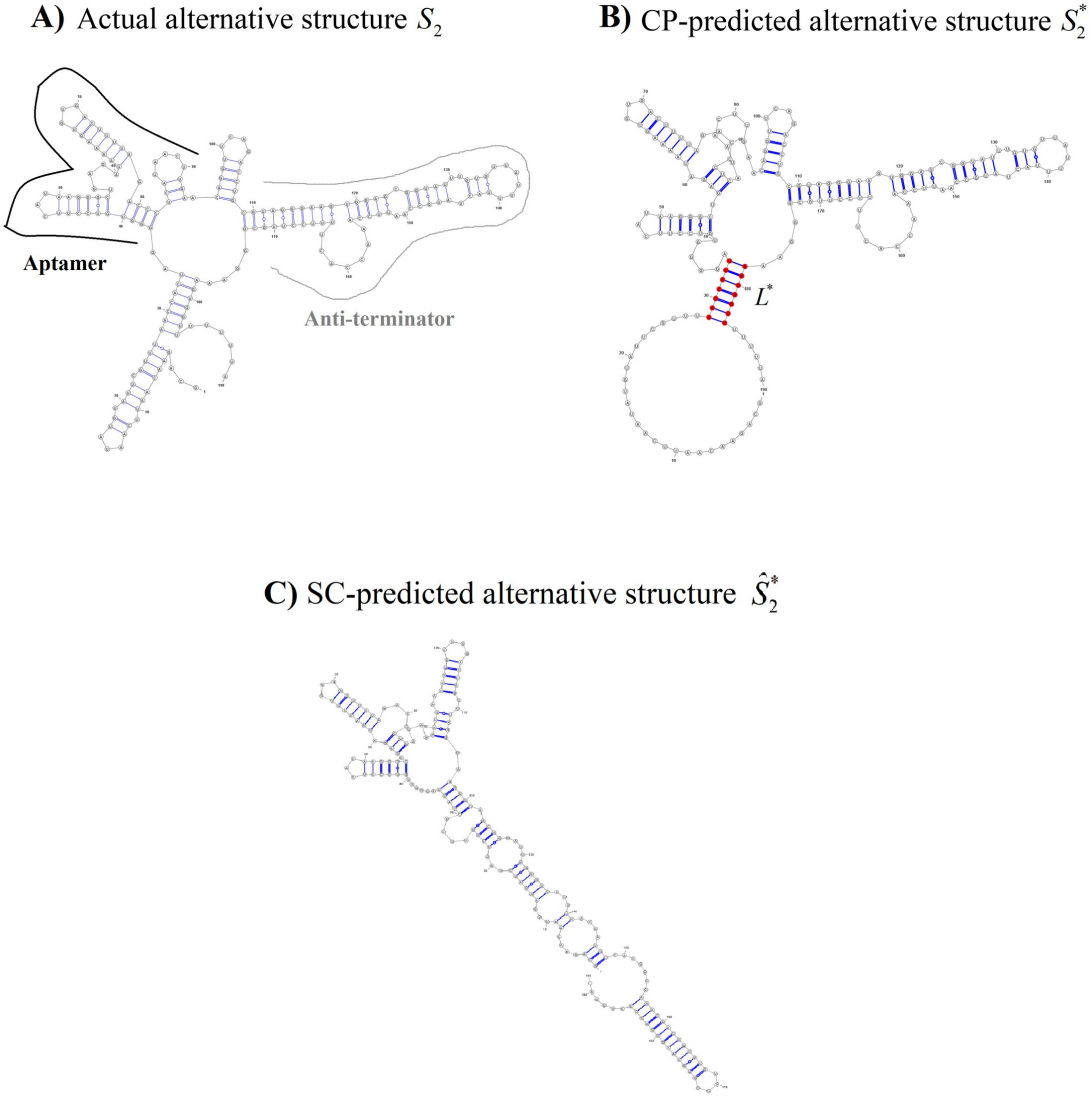
Prediction of the alternative structure of *tenA* TPP riboswitch. Sequence length in the Barsacchi Dataset was 190nt. A) The actual alternative structure *S*_2_ (here, the unbound state), with the approximate locations of the aptamer substructure and the anti-terminator stem as indicated. B) The CP-predicted alternative structure $S_{2}^{*}$. The stem seed L* = {28…34·178…184} is shown in red. C) The SC-predicted alternative structure ${\hat{S}}_{2}^{*}$ (See Materials and methods for details).

Empirical results motivated our defaults for L* and *τ*. Our choice of *τ* provides some insight into our methods. Briefly, for *τ* = 0 the excluded set $E^{\left(\tau =0\right)}=\left\{\left[i\cdot j\right]:\left[i\cdot j\right]\in S^{*}\right\}$ is precisely the set of base pairs in $S_{1}^{*}$. Empirically, $E^{\left(\tau =0\right)}$ often excluded too few base pairs to make $S_{2}^{*}\left(\tau =0\right)$ markedly different from $S_{1}^{*}$. We selected $S_{2}^{*}\equiv S_{2}^{*}\left(\tau =5\right)$ as our default for alternative structure prediction. Fig 2 illustrates the actual alternative structure (here, unbound structure) of *tenA* TPP and its corresponding predictions. Fig 2A illustrates the actual alternative structure *S*_2_ with its aptamer region and anti-terminator stem. Fig 2B illustrates $S_{2}^{*}$, with the stem seed L* shown in red.

The established approach for predicting the two structural states of a riboswitch uses a Sampling-Clustering (SC) method. Every SC computation in this article followed standard protocol by sampling RNA structures at many different temperatures. Fig 2B illustrates alternative structure prediction under SC (see the Materials and methods section for details of our SC implementation). As we can see, for this elongation of TPP riboswitch, the CP method recognizes critical substructures of the alternative structure better than the SC method.

**Fig 3:**
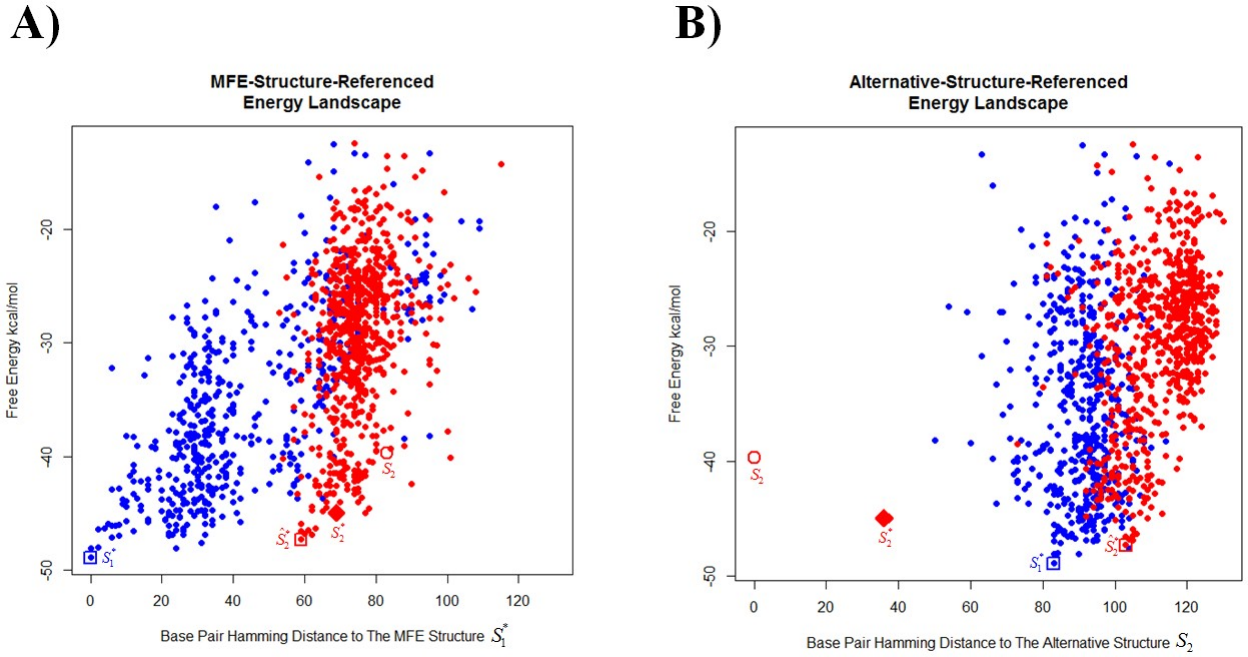
Energy landscape of *tenA* TPP. The open blue square represents the computed MFE structure $S_{1}^{*}$; the open red circle, the actual alternative structure *S*_2_; the solid red diamond, the CP-predicted alternative structure $S_{2}^{*}$; and the open red square, the SC-predicted alternative structure ${\hat{S}}_{2}^{*}$. Blue and red dots represent samples of the MFE-containing and alternative clusters, respectively (see the Materials and methods section for details on our SC implementation). A) A standard depiction of an energy landscape, where the MFE structure $S_{1}^{*}$ provides the reference structure at distance 0. B) The Alternative-Structure-Referenced (ASR) energy landscape, where the actual alternative structure *S*_2_ provides the reference structure at distance 0. Some important base-pair Hamming distances can be read directly from Fig 2A and 2B: $d_{H}\left\{S_{2}\text{,}S_{1}^{*}\right\}=83$ (from both 2A and 2B); $d_{H}\left\{{\hat{S}}_{2}^{*}\text{,}S_{1}^{*}\right\}=59$ and $d_{H}\left\{S_{2}^{*}\text{,}S_{1}^{*}\right\}=69$ (from 2A). $d_{H}\left\{{\hat{S}}_{2}^{*}\text{,}S_{2}\right\}=103$ and $d_{H}\left\{S_{2}^{*}\text{,}S_{2}\right\}=36$ (from 2B).

Fig 3 displays the energy landscape for the *tenA* TPP example. Fig 3A displays a standard depiction of an energy landscape, where the X-axis gives base-pair Hamming distance to $S_{1}^{*}$ for each sampled structure *S*. Because our main interest is structural prediction of *S*_2_, Fig 3B depicts our Alternative-Structure-Referenced (ASR) energy landscape, where the X-axis gives base-pair Hamming distance of each sampled structure *S* to *S*_2_. Structural dissimilarities of predictions to *S*_2_ are better illustrated in the ASR energy landscape than in the standard energy landscape.

### Conditional-Probability gave base-pair prediction performance superior to Sampling-Clustering

To assess predictive accuracy of CP and SC, a subset of the Barsacchi dataset provided 12 riboswitches with known actual alternative structures *S*_2_ (our “Barsacchi Structural dataset”). Within the Barsacchi Structural dataset, we compared *S*_2_, our gold standard, to three predicted alternative structures: ${\hat{S}}_{2}^{*}$ (from SC), $S_{2}^{*}\left(\tau =0\right)$ (from CP, with *τ* = 0), and $S_{2}^{*}$ (from CP, with default *τ* = 5). For the three predicted alternative structures ${\hat{S}}_{2}^{*}$, $S_{2}^{*}\left(\tau =0\right)$, and $S_{2}^{*}$, we calculated their sensitivity and specificity values (i.e., total-SEN and total-PPV in [74]), along with the F measure, another measure of prediction accuracy [75]. Table 1 gives the overall performance of the three predictions (S2 Table gives the details of performance for each riboswitch). The total-SEN, total-PPV, and F measure ordered the performance of the three predictions consistently: $S_{2}^{*}\left(\tau =0\right)$ was slightly inferior to ${\hat{S}}_{2}^{*}$, but $S_{2}^{*}$ was noticeably superior to them both.

**Table 1:** Alternative-structure prediction performance in the Barsacchi Structural dataset.

Predicting the alternative structure via	total-SEN (%)	total-PPV (%)	F measure	Time
SC	43.0	37.4	0.100	00:18:23^x^
CP under zero threshold	36.2	31.1	0.084	00:00:01
CP under the default threshold	51.3	46.1	0.121	00:00:01

The text explains the performance measures. The (parallelized) computational time reflects the longest sequence in the dataset, the *LysC* Lysine riboswitch (243nt). The SC time (marked x) does not include the clustering computation. All computations were performed on a 12 core Intel(R) Xeon(R) 2.93GHz CPU. The performance of SC ${\hat{S}}_{2}^{*}$, CP under zero threshold $S_{2}^{*}\left(\tau =0\right)$, and CP under the default threshold $S_{2}^{*}$ are shown below.

### Conditional-Probability was about 1000 times faster than Sampling-Clustering

In computational speed on the Barsacchi Structural dataset, CP was more than three orders of magnitude faster than SC (see Table 1). Computations were parallelized, so the longest sequence in the dataset, here the *LysC* Lysine riboswitch (243nt), determined the speeds. Typical CP runtimes increased with increasing dissimilarity thresholds, but even a dissimilarity threshold of half the length of the sequence, *τ* = 122, only increased the CP runtime to 6 seconds. Our SC runtimes included the sampling step, but not the clustering step. The whole CP computation of $S_{2}^{*}$ (i.e., under default value *τ* = 5) took around a second, whereas the sampling step of SC alone took more than 18 mins.

### Riboswitch classification

**Fig 4:**
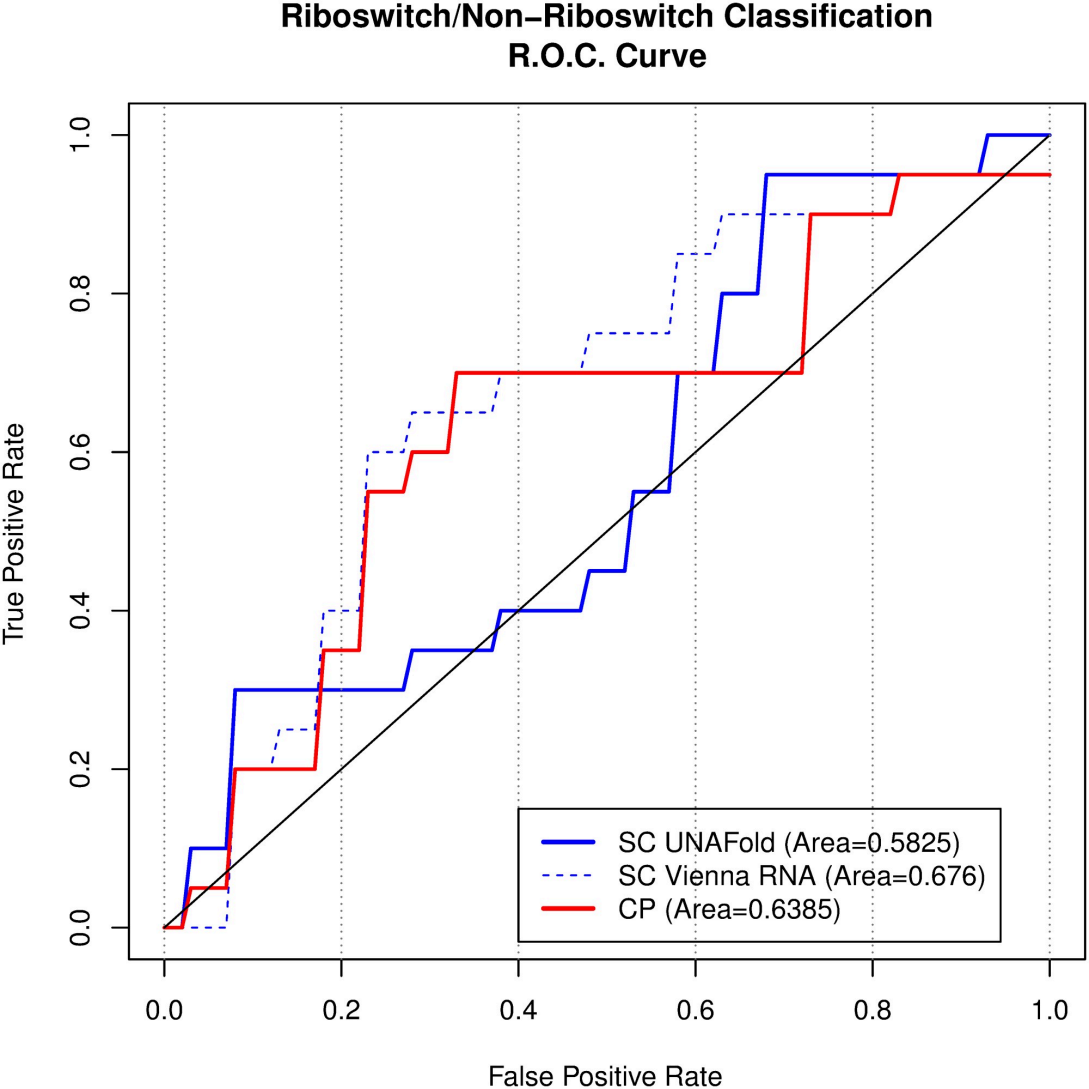
ROC. curves for SC- and CP-based riboswitch classifiers. The blue and red lines correspond to SC- and CP-based classifiers. The legend gives the ROC value, the area under the curves for the two classification methods. Calculations were done at 37°C for all three classifiers.

To compare how well CP and SC can classify RNA sequences into riboswitch or non-riboswitch, we used the full Barsacchi Dataset (20 riboswitches vs. 20 non-riboswitches). SC classified each sequence according to the average silhouette value of the two clusters of samples; CP, according to normalized seed length (see the Materials and methods section). Compared to non-riboswitches, riboswitches tend to have both a higher average silhouette value [54] and normalized seed length (4.00bp for riboswitches; 3.50bp for non-riboswitches). Fig 4 displays the corresponding ROC curves for SC (in blue) and CP (in red).

We tuned the SC computations more extensively in riboswitch classification than in other parts of our study, exploring multiple temperatures or 37°C alone to generate the samples, since performance of SC can highly depend on sampling parameters. To make our comparison to the best SC performance, we included Vienna RNA results, which had been previously used on Barsacchi Dataset. SC classification performance was better at 37°C alone (area under curve 0.5825, solid blue in Fig 4) than at multiple temperatures (area under curve 0.455) under the UNAFold software. Area under curve was higher using Vienna RNA at 37°C alone (0.676, dashed blue in Fig 4). In our hands and on the Barsacchi Dataset, the performance of the CP-based classifier was comparable to SC under Vienna RNA sampling when differentiating riboswitches from non-riboswitches (0.6385 for CP compared to 0.676 for SC, difference 0.0375±0.1227 with two-tailed p-value 0.76 [76]), and higher compared to SC under UNAFold (0.5825).

### The seeds L* pointed to critical substructures in the purine riboswitch family

**Fig 5:**
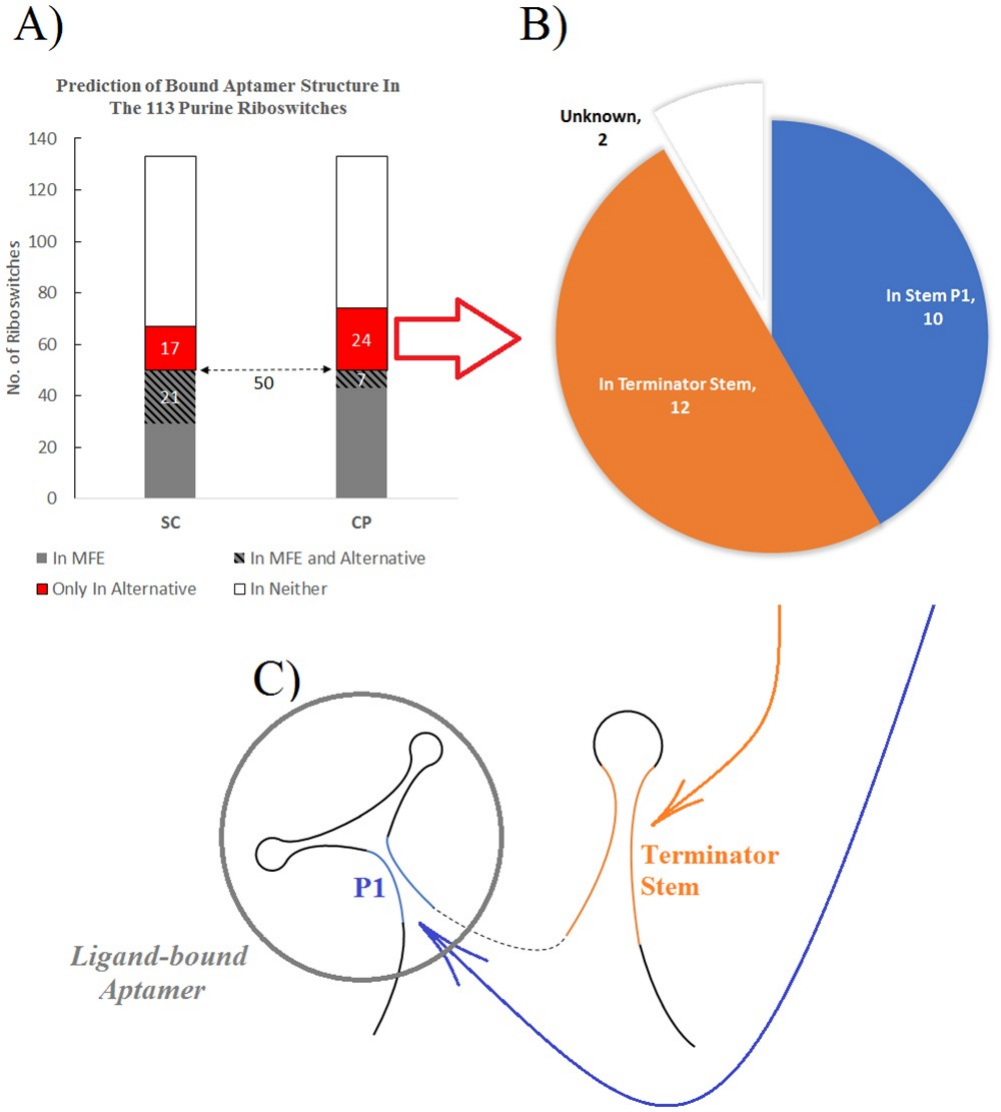
Alternative structure prediction in the purine riboswitch dataset. We examined the predicted structures $S_{1}^{*}$, $S_{2}^{*}$, and ${\hat{S}}_{2}^{*}$ for Stem P1, a substructure of the bound aptamer. A) The number of predicted structures containing Stem P1. The left bar corresponds to SC (which predicts $S_{1}^{*}$ and ${\hat{S}}_{2}^{*}$); the right bar, to CP (which predicts $S_{1}^{*}$ and $S_{2}^{*}$). B) The location of the stem seed L* for the 24 sequences where CP predicted the bound aptamer in $S_{2}^{*}$ but not $S_{1}^{*}$. C) The schematic structure of a typical ligand-bound purine riboswitch.

The Materials and Methods section details the construction of our Purine Riboswitch dataset from Rfam. The Purine Riboswitch dataset contained 133 sequences, each of length about 200nt, constructed with the intent that the sequences were long enough to fold into both unbound and purine-bound structures. Rfam provides a consensus structure for the ligand-bound aptamer, which we mapped onto each of the 133 sequences. Stem P1 of the ligand-bound aptamer (Fig 5C) is fully formed only if the aptamer has bound a purine nucleotide, and not otherwise [77]. As above, we predicted $S_{1}^{*}$, $S_{2}^{*}$, and ${\hat{S}}_{2}^{*}$ for each sequence in the dataset and examined whether the predicted structure contained Stem P1, a substructure of interest in the bound state of a purine riboswitch. (Here, the comparison of the CP and SC methods must consider the computed MFE structure $S_{1}^{*}$, because it can correspond to either of the bound and unbound structures.) To describe Fig 5A, of 133 sequences, $S_{1}^{*}$ (predicted by both CP and SC) contained Stem P1 for 50 sequences. Of the 50 sequences where $S_{1}^{*}$ contained Stem P1, $S_{2}^{*}$ (predicted by CP) contained Stem P1 for 7 sequences; and ${\hat{S}}_{2}^{*}$ (predicted by SC), for 21 sequences. Of the remaining 83 = 133–50 sequences where $S_{1}^{*}$ did not contain Stem P1, $S_{2}^{*}$ (predicted by CP) contained Stem P1 for 24 sequences, and ${\hat{S}}_{2}^{*}$ (predicted by SC) contained Stem P1 for 17 sequences.

For the 24 sequences where CP predicted Stem P1 in $S_{2}^{*}$ but not in $S_{1}^{*}$, we investigated the location of the stem seed L*. Interestingly, of the 24 sequences, L* was almost identical to Stem P1 in 12 sequences, and in 10 of the remaining sequences, L* was distant from the aptamer and near the terminator stem. In those cases, the CP stem seed L* contained many consecutive base pairs overlapping the actual terminator stem (see Fig 5B). In the remaining 2 cases, L* had no clear biological interpretation. In summary, therefore, in 22/24 purine riboswitches where Stem P1 of the aptamer was in $S_{2}^{*}$ but not $S_{1}^{*}$, the stem seed L* pointed to a regulatory substructure in the purine riboswitch.

### A case study: How adjustable parameters in CP affect prediction accuracy

The generalized CP prediction $S_{2}^{*}$ of alternative structure has only a few adjustable parameters: dissimilarity threshold *τ* (default: 5), temperature *T* (default: 37°C), *type-of-seed* (default: stem seed L*), and iteration number *i* (default: 2). The Materials and Methods section describes the parameters in detail. Our default parameters (particularly for *τ* and L*) were tuned empirically by their predictions on the Barsacchi Structural dataset, but the sparsity of the data prevented methodical optimization (and any formal separation of training and test datasets). The following uses the *xpt* Guanine riboswitch [2, 57], 162nt long in the Barsacchi Structural dataset, as an anecdotal example of how $S_{2}^{*}$ varies with the parameters.

**Fig 6:**
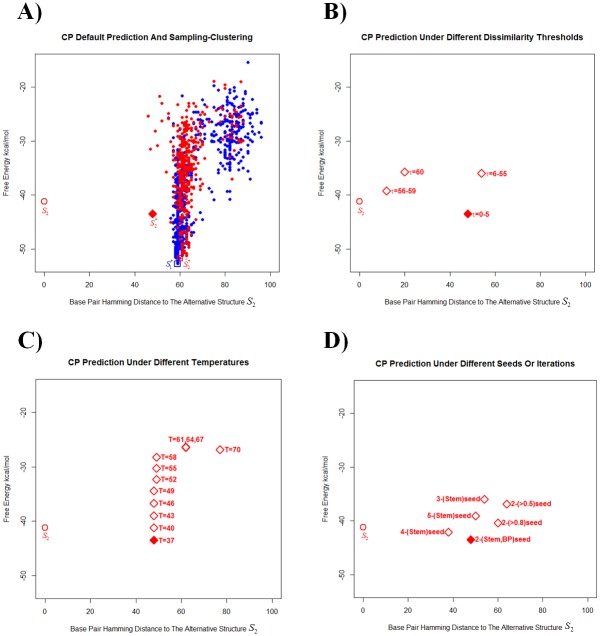
Alternative-Structure-Referenced (ASR) energy landscape of *xpt* Guanine riboswitch. The sequence length was 162nt, directly from the Barsacchi dataset. In each subfigure, the open red circle on the left represents *S*_2_. In Fig 5B–5D, the solid diamond represents CP prediction under default parameters; and the empty diamonds, CP prediction under non-default parameters. A) The solid red diamond represents the CP-predicted alternative structure $S_{2}^{*}$; and the open red square, the SC-predicted alternative structure ${\hat{S}}_{2}^{*}$. Blue and red dots represent samples of the MFE-containing and alternative clusters, respectively (see Materials and methods for details on the Sampling-Clustering procedure). B) CP-predicted alternative structures $S_{2}^{*}$ under different dissimilarity thresholds *τ* = 0…60. If predictions were identical, they are shown with a label indicating a range for *τ*. C) CP-predicted alternative structures $S_{2}^{*}$ under different temperatures *T* from 37°C to approximate melting temperature 70°C. The free energy of any fixed structure varies with temperature, and the underlying RNA structure remained fixed for temperatures 37≤*T*≤49, 50≤*T*≤58, and 61≤*T*≤67. D) CP-predicted alternative structures $S_{2}^{*}$ under different seeds and iterations. The initial number in the label of each point gives the iteration *i* = 2…5. The seed was either a stem seed L*, a base-pair seed (the base pair with highest conditional probability, denoted by “BP”), or all base pairs with conditional probability higher than a threshold (either 0.5 or 0.8). For iteration number *i* = 2, the CP-predicted $S_{2}^{*}$ from stem and base-pair seeds were identical: hence, its label “2-(stem,BP)-seed”.

In Fig 6, ASR energy landscapes display the actual unbound structure *S*_2_ of *xpt* Guanine on their left, as a standard for comparing other structures. For example, Fig 6A displays structural samples from the Boltzmann distribution, along with the predicted alternative structures $S_{2}^{*}$ (CP) and ${\hat{S}}_{2}^{*}$ (SC). Fig 6B–6D vary *τ*, *T*, the *type-of-seed*, or the number of iterations, showing the effects of changing parameters on CP predictions of the alternative structure. Fig 6B shows that the 61 dissimilarity thresholds *τ* = 0…60 produced only five distinct predictions $S_{2}^{*}$. Fig 6C shows that the 12 temperatures *T* = 37,40,43,46,49,52,55,58,61,64,67,70 (from 37°C to an approximation of the melting temperature 69.8°C in 3°C increments) produced only four distinct predictions $S_{2}^{*}$. We also could have folded $S_{2}^{*}$ around a seed structure other than a stem. Fig 6D shows different predictions resulting from: (1) various seeds, e.g., base pairs with conditional probabilities higher than 0.5 and 0.8, and stems of various fixed lengths; and (2) various numbers of iterations of the exclusion-conditional probability calculation (e.g., predictions $S_{3}^{*}$, $S_{4}^{*}$, and $S_{5}^{*}$ from 3, 4, and 5 iterations using a stem seed: see the Materials and methods subsection, Generalization by iteration number). Overall, base-pair Hamming distance $d_{H}\left\{S_{2}^{*},S_{2}\right\}$ from $S_{2}^{*}$ to *S*_2_ took very few different values as the individual parameters varied (i.e., relatively few distinct predicted structures $S_{2}^{*}$ appeared as the parameters varied).

## Discussion

Computational identification of RNA switches and substructures solely from sequence could elucidate biological control mechanisms in many species, and successful identification of switches could accelerate the discovery of new sensors and control mechanisms, particularly in bacteria and other prokaryotes. For an RNA switch (or more specifically, for a typical riboswitch), predicting an alternative biologically functional structure can be challenging. Such predictions hold the key to understanding many regulatory mechanisms, however. Although riboswitches are a very diverse subset of RNA switches, there is not enough experimentally verified data for optimizing a set of parameters of a universal RNA Switch Predictor.

### Conditional-Probability and Sampling-Clustering produced comparable predictions in the Barsacchi dataset [54]

We used the Barsacchi dataset [54] as a benchmark mainly to have independently selected sequence lengths for our study. In its first step, the CP prediction of alternative structure excludes from the energy landscape those base pairs belonging to a metastable state, here, the MFE structure. Then, it explores the remaining energy landscape for stable substructures. The prediction has relatively few adjustable parameters, so we gave them a preliminary default tuning with a structural subset of the Barsacchi dataset.

We predicted the CP alternative structure using only the Boltzmann ensemble at 37°C. In our hands, compared to a implementation of the current SC procedure [18, 44, 54], which included sampling from many temperatures, CP predicted alternative structures better than SC (Table 1). Thus, although the sparsity of relevant data required us to use the Barsacchi dataset [54] for both training and testing, Table 1 suggests (at the very least) that Conditional-Probability (CP) predictions can be competitive with current Sampling-Clustering (SC) methods for predicting the alternative structure. In fact, Table 1 gives very conservative performance values for CP, because only the bound structure was available for three of the twelve riboswitches (see S2 Table). For these three riboswitches, the MFE prediction $S_{1}^{*}$ corresponded to the actual bound state *S*_2_, so the CP-predicted alternative state $S_{2}^{*}$ was different, lowering CP prediction performance. Nonetheless, prediction performance of CP under the default dissimilarity threshold was still higher than that of SC.

### Conditional-Probability vs Sampling-Clustering produced comparable predictions in the purine riboswitch dataset

To avoid cherry-picking in our Purine Riboswitch dataset, a purine riboswitch family of 133 Rfam sequences, we fixed all sequence lengths at 200nt. Fig 5A again suggests that CP predictions are also competitive with SC predictions in the Purine Riboswitch dataset. In fact, CP confined predictions of the Stem P1 in the bound aptamer substructure more to the alternative structure than SC (cf., 7/50 under CP to 21/50 under SC). This relatively exclusive prediction is probably desirable in the alternative structure predictor, because the ligand-bound aptamer is exclusive to only one of the two functional structures of a purine riboswitch. Overall, however, the CP default parameter predictions made at a single temperature of 37°C again had comparable performance to an SC prediction at multiple temperatures.

### Conditional-Probability was more than 1000 times faster than Sampling-Clustering

The computed free energy of the bound structure of a riboswitch does not include ligand binding energies, and consequently it may be arbitrarily high. Regardless, CP prediction always has a polynomial time-complexity. Denote sequence length by *n* and the dissimilarity threshold by *τ*. CP prediction has five steps (each step is followed by its time-complexity): MFE prediction (*O*(*n*^4^)); excluded set determination (τ^2^*O*(*n*)); conditional probability calculation (*O*(*n*^3^)); seed selection (*O*(*n*^2^)); and prediction of the alternative structure by constrained energy minimization (*O*(*n*^4^)). Thus, the total computational time *t*_*cp*_(*n*,*τ*) = *τ*^2^*O*(*n*)+*O*(*n*^4^). In contrast, SC prediction can be sensitive to the computed free energy of the bound structure, because folding probabilities exponentially decrease with energy. The time-complexity of exhaustive sampling is exponential in both the sequence length *n* and the maximum energy explored. The complexity becomes more manageable under statistical sampling, but then it depends on the number of samples (*s*) as well as *n*. A typical SC procedure such as ours consists of three steps: MFE prediction (*O*(*n*^4^)); sampling (*sO*(*n*^2^)); and clustering (*s*^2^O(*n*)). Thus, the total computational time *t*_*sc*_(*n*,*s*) = *O*(*n*^4^)+*sO*(*n*^2^)+*s*^2^*O*(*n*). Thus, the time complexities of the two procedures have a term *O*(*n*^4^). The SC prediction requires more sampling as the sequence lengthens, burdening computer memory and increasing the computation times accordingly. CP predictions, on the other hand, require more memory as *τ* increases. We generally expect CP predictions to be much faster than SC even for RNA sequences of moderate length (e.g., *n* equaling a few hundred).

Typically, riboswitch lengths are moderate, so the sample sizes *s* necessary for effective prediction remain feasible. To compare the speed of CP and SC predictions, Table 1 shows that despite its far superior speed, even when *t*_*sc*_ excludes the time for clustering the samples, CP can still outperform SC in predictive accuracy (e.g., for *LysC* Lysine, *t*_*cp*_(*n* = 243,*τ* = 5) equals about 1 second; *t*_*sc*_(*n* = 243,*s* = 1200), more than 18 minutes). The time for CP alternative structure prediction does increase as *τ* increases from its default value *τ* = 5, but it still remains far faster than SC prediction (e.g., *t*_*cp*_(*n* = 243,*τ* = 122) equals only 6 seconds). To summarize, in our hands CP predictions were about 1000 times faster than SC predictions, with at least comparable prediction accuracy.

### The effect of the dissimilarity threshold *τ* on CP predictions

CP generates a seed around which we fold an alternative structure. Loosely, when generating the CP seed, the dissimilarity threshold *τ* effectively excludes the formation of base pairs “close to” the MFE structure. It therefore enforces topological differences between alternative and MFE structures (see S1 File, which relates *τ* to the Relaxed Base Pair score ρ_*t*_(*S*_1_,*S*_2_) between structures [73]). The threshold *τ* = 0 excludes all base pairs in the MFE structure, and as *τ* increases, the exclusion procedure progressively excludes more and more base pairs. Empirically, our default *τ* = 5 had a better performance than simply excluding MFE base pairs with *τ* = 0 (see Table 1). The CP prediction accuracy was relatively robust near the default *τ* = 5, i.e., *τ* close to *τ* = 5 predicted similar alternative structures (e.g., 0≤*τ*≤5 for *xpt* Guanine in Fig 6B). In fact, for the few sequences in our Barsacchi Structural dataset, predictions often did not change much for small values of *τ*. On the other hand, some high values of *τ* (56≤*τ*≤59) improved the prediction accuracy for *xpt* Guanine dramatically (see Fig 6B). Unfortunately, attempts to tune *τ* through intricate dependencies on (e.g.) sequences or conditional probabilities did not change prediction accuracies much. Large *τ* values sometimes improved predictions dramatically for individual riboswitches, but unfortunately no general and systematic theme of improvement became apparent.

### The effect of the seed on CP predictions

The seed is another adjustable CP parameter. After excluding base pairs close to the MFE structure from the energy landscape (see above), a seed is merely a substructure that occurs frequently in the remaining structures. Empirically, the longest bulge-containing consensus stem L* made a good default seed for alternative structure prediction. We tested other seeds (e.g., seeds consisting of a single base pair), but the corresponding alternative structure predictions were generally less accurate (see the anecdotal case study in Fig 6D). Seeds with too many base pairs often produce poor predictions, because MFE and alternative structures often share many of the aptamer substructures.

### Riboswitches tend to have longer stem seeds than non-riboswitches

A riboswitch classifier can use normalized seed length, because riboswitches tend to yield longer stem seeds than RNAs with a single structure. With our data and under the CP method, the normalized seed length of a riboswitch is 4.00bp; of a non-riboswitch, 3.50bp (see the Results section). Thus, a riboswitch of length 100nt has a stem seed of expected length 8bp (4.00×log_10_ = 8); a non-riboswitch of the same length; 7bp. The use of seed length in classification takes advantage of the notion that base pairs may have evolved to stabilize an alternative structure in riboswitches [45] while this evolutionary pressure does not exist in non-switching RNAs. In many purine riboswitches, the stem seed pointed to critical control substructures exclusive to the bound structure (Fig 5B). Although we did not explore other riboswitch families, it is interesting to speculate that if a folding pathway does not contain the MFE structure, a CP seed may often point to important regulatory substructures. Although different regulatory mechanisms of different RNA switches may influence the *type-of-seed*, for the purine riboswitches at least, biologically functional substructures often included our stem seed.

### The effect of other parameters on CP predictions

The CP temperature parameter *T* may be particularly useful for predictions in riboswitches with thermosensory functions. Because RNA thermosensors lay beyond our purview, however, the present article relied on the default temperature 37°C for CP prediction of alternative structures (note that predictions from sampling always sampled at several temperatures, however). The use of other temperatures in CP prediction did not change prediction accuracy dramatically. We also considered iterating our three-step CP prediction process: (1) exclude base pairs (2) determine a stem seed; and (3) find the lowest free-energy structure containing the stem seed (see the Materials and methods section for details). The threshold parameter *τ* seemed to offer a more gentle and graduated exclusion of base pairs than iteration, however. Iteration may prove useful for riboswitches with more than two metastable structures, but here, it only improved CP alternative structure prediction very occasionally (see Fig 6D, where the iterated predicted structure $S_{4}^{*}$ is closer to the actual alternative structure *S*_2_ than the default prediction $S_{2}^{*}$).

## Conclusion

In this article, we use *exact* conditional base-pair probabilities to predict the alternative structure of RNA switches. Our approach selects base pairs associated with high conditional probabilities, after it excludes substructures in the primary metastable structure (here, the MFE structure). Conditioning on exclusion improves the chance that an exact (McCaskill) probability calculation finds base pairs in an alternative structure. Within the limitations imposed on our ROC tests by available data, Conditional-Probability (CP) computations had classification accuracy comparable to Sampling-Clustering (SC) computations. In contrast, however, the results for computational speed were not at all tentative: CP was more than 1000 times faster than SC, its speed making it a much more promising as a predictor of alternative structures in computationally demanding settings like genomic RNA.

CP predictions have very few adjustable parameters. Specifically, our dissimilarity threshold parameter *τ* varies the severity with which metastable substructures are excluded from predictions. Although available data is insufficient to optimize *τ* methodically, our default choice *τ* = 5 was effective enough to render CP at least comparable to SC. Moreover, our tests suggest that varying *τ* offers a novel and computationally efficient method of traversing the energy landscapes to find metastable structures. We also showed that another important CP parameter, the seed, could detect functional substructures in riboswitches with regulatory functions as well as being able to classify riboswitches with similar performance to the more computationally costly sampling and clustering of the RNA energy landscape.

## Availability

Our CondAlt source code for predicting alternative structures is publicly available for download at https://go.usa.gov/xRu79. The data used in the first dataset (Barsacchi) is available in S2 File and also publicly available in [54]. The data used in the second dataset (Purine aptamers) were taken from Rfam. Please refer to Material and Methods for further details.

## Supporting information

S1 Table Barsacchi riboswitch dataset. The 20 Riboswitches and their corresponding lengths.
(PDF)

S2 Table Sensitivity and Positive-Predictive-Values (PPV) of the Barsacchi Structural riboswitch set. (PDF)

S1 File The relationship between Relaxed Base Pair score and dissimilarity threshold. (PDF)

S2 File Barsacchi datasets. (PDF)
