# Supplementary material for: Structural prediction of RNA switches using conditional base-pair probabilities
Source: PLoS One. 2019 Jun 12;14(6):e0217625. doi: 10.1371/journal.pone.0217625 (PMC6561571; doi:10.1371/journal.pone.0217625)
Supplement: S1 Table — The 20 Riboswitches and their corresponding lengths. (PDF) [file pone.0217625.s001.pdf]

Supporting Information

S 1 Table. Barsacchi riboswitch dataset. The 20 Riboswitches and their corresponding lengths are available in the following table. The first set of 12 structures where the bound state structure was available is referred to as the Barsacchi structure dataset. The set of first 9 riboswitches both MFE and alternative structures are available and in the remaining three, only the bound state is available.

ID	Riboswitch	No. of Structures Available	Length
1	mgtE Mg	Both	213
2	tenA TPP	Both	190
3	ECOL thi MTPP	Both	151
4	Add Adenine	Both	147
5	Xpt Guanine11	Both	162
6	ydhL pbuE Adenine	Both	107
7	VEGFA	Both	125
8	lysC Lysine	Both	244
9	BSUBT yitI SAM	Both	170
10	thiC TPP A. thaliana	One	151
11	folT THF	One	161
12	metH SAH	One	111
13	ECOL Moco moaA	Neither	134
14	GEMM CDA	Neither	127
15	FNUC PREQ1	Neither	69
16	B. cereus crc Flouride	Neither	79
17	ydaO ATP	Neither	203
18	btuB B12	Neither	244
19	metA SAMII	Neither	186
20	PH alx	Neither	212
