# Supplementary material for: Structural prediction of RNA switches using conditional base-pair probabilities
Source: PLoS One. 2019 Jun 12;14(6):e0217625. doi: 10.1371/journal.pone.0217625 (PMC6561571; doi:10.1371/journal.pone.0217625)
Supplement: S2 Table — (PDF) [file pone.0217625.s002.pdf]

Supporting Information

S 2 Table. Sensitivity and Positive-Predictive-Values (PPV) of the Barsacchi Structural riboswitch set. Alternative structure predictions of the three methods, CP ($\tau = 0$), CP ($\tau = 5$), and Sampling-Clustering (SC) were compared to actual alternative structures of the riboswitches provided in Barsacchi Structural dataset. Alternative structure of VEGFA, shown by *, was selected as the one not predicted by the MFE structure. For sequences shown with **, only one structure (the bound structure) was available in the literature. Their corresponding bound structure was partially predicted by MFE structure prediction (not shown).

	**CP ($\tau = 0$)**	**CP ($\tau = 0$)**	**CP ($\tau = 5$)**	**CP ($\tau = 5$)**	**SC**	**SC**
**Riboswitch**	**SEN (%)**	**PPV (%)**	**SEN (%)**	**PPV (%)**	**SEN (%)**	**PPV (%)**
mgtE Mg	52.63	49.18	82.46	81.03	92.98	84.13
tenA TPP	18.75	21.05	73.44	94	28.12	31.03
ECOL thi MTPP	46.67	44.68	46.67	44.68	55.56	55.56
Add Adenine	51.22	45.65	51.22	45.65	14.63	30
Xpt Guanine11	56.41	52.38	56.41	52.38	43.59	36.96
ydhL pbuE Adenine	24	25	24	25	24	17.14
VEGFA*	0	0	61.54	57.14	0	0
lysC Lysine	58.33	42.68	41.67	33.78	18.33	14.29
BSUB yitI SAM	92.5	84.09	92.5	84.09	47.5	38.78
thiC TPP A. thaliana**	60	52.5	0	0	60	51.28
folT THF**	0	0	0	0	55.17	34.78
metA SAH**	0	0	0	0	100	32.35
