# Supplementary material for: Structural prediction of RNA switches using conditional base-pair probabilities
Source: PLoS One. 2019 Jun 12;14(6):e0217625. doi: 10.1371/journal.pone.0217625 (PMC6561571; doi:10.1371/journal.pone.0217625)
Supplement: S1 File — (PDF) [file pone.0217625.s003.pdf]

Supporting Information

S 1 File. The Relationship between Relaxed Base Pair Score and Dissimilarity Threshold τ

Here, we show that under dissimilarity threshold $\tau = \tau_0$, the predicted alternative structure is guaranteed to have a non-zero Relaxed Base-Pair Score, under relaxation parameter t, so long as $t \le \tau_0 + 1$. For instance, given the default prediction under $\tau = 5$, $\rho_{t \le 6}\left(S_1^*, S_2^*\right) > 0$.

The Relaxed Base Pair (RBP) score with relaxation parameter t is $\rho_t\left(S_1, S_2\right) = \min\left\{m \in \mathbb{Z} \mid m \ge 0, \Delta_k \le tm \text{ if } k > m\right\}$, where Δ_k are sorted δ_{bs} distances in descending order [1]. Given predictions S_1^* and S_2^*, the base pair(s) corresponding to the seed have $\Delta \ge \tau + 1$, making $\max \Delta_i \ge \tau + 1$.

For $t = \tau = 0$:

In this case, $\max \Delta_i \ge 0 + 1$. RBP score is equal to the number of non-zero distances, i.e., the Hamming distance. $\rho_0\left(S_1^*, S_2^*\right) = d_H\left\{S_1^*, S_2^*\right\} > 0$.

For $0 < t \le \tau$:

In this case, $\max \Delta_i > t$. RBP score $\rho_t\left(S_1^*, S_2^*\right) > 1$, since neither of $m = 0$ or $m = 1$ satisfy the minimization. The RBP score will satisfy for some $m > 1$.

For $t = \tau + 1$:

In this case, $\max \Delta_i \ge t$. RBP score can vary depending on Δ_i values: $\rho_{t=(\tau+1)}\left(S_1^*, S_2^*\right) \ge 1$. For a boundary condition of $\max \Delta_i = t$, where all base pairs in S_2^* including the seed base pair(s) are only $\tau + 1$ distant or less from S_1^*, RBP score is $\rho_{\tau+1}\left(S_1^*, S_2^*\right) = 1$, since the minimization is satisfied for $m = 1$.

For $t > \tau + 1$:

In this case, can vary depending on Δ_i values: $\rho_t\left(S_1^*, S_2^*\right) \ge 0$.
