# Supplementary material for: Structural prediction of RNA switches using conditional base-pair probabilities
Source: PLoS One. 2019 Jun 12;14(6):e0217625. doi: 10.1371/journal.pone.0217625 (PMC6561571; doi:10.1371/journal.pone.0217625)
Supplement: S2 File — (PDF) [file pone.0217625.s004.pdf]

Supporting Information

S 2 File. Barsacchi dataset. 20 Riboswitch sequences and their corresponding structures. 20 non-riboswitches and their corresponding sequences (negative control). Data courtesy of Barsacchi et al.

```
>1 mgtE Mg
ACTTCGTTAGGTGAGGCTCCTGTATGGAGATACGCTGCTGCCCAAAAATGTCCAAAGACGCCAATGGGTCAACAGAAATCATCGAC
ATAAGGTGATTTTTAATGCAGCTGGATGCTTGTCCTATGCCATACAGTGCTAAAGCTCTACGATTGAAGGCGCCCGCACGCTTTTT
TTGCCGTGCTTCTTTCACCTTCAATCCCGAAGGCTTTTTTT
.(((((.(((((..(((..((((((((......(((((.(((((....((((....))))....)))))....((((((((((...
....))))))))))...))))).((((....))))....)))))))).)))...)).))).(((((((((.(...((((((.....
..).))))))......).)))))))))).))))).........
...((((..(((..(((..((((((((......(((((.(((((....((((....))))....)))))....((((((((((...
....))))))))))...))))).((((....))))....)))))))).)))...)))..))))...........((((((.....
..).))))).........(((((......))))).........
>2 tenA TPP
GCAGAACAATTCAATATGTATTCGTTTAACCACTAGGGGTGTCCTTCATAAGGGCTGAGATAAAAGTGTGACTTTTAGACCCTCAT
AACTTGAACAGGTTCAGACCTGCGTAGGGAAGTGGAGCGGTATTTGTGTTATTTTACTATGCCAATTCCAAACCACTTTTCCTTGC
GGGAAAGTGGTTTTTTTA
.........(((..((((((...((((((((.....(((((((((...))))))).....((((((((...))))))).)))))(((.
....)))...)))).)))).)))))))..)))..((((.(((((..(((......))).)))))..))))(((((((((((((((....
)))))))))))))).....
...(((((((.((...)).))).))))(((((((..((((((((((...))))))).....((((((((...))))))).)))))(((.
....))).(((((....)))))((((((((((((((.(((((..(((......))).)))))..)))))).......)))))))))
)....))))))).......
>3 ECOL Moco moaA
GGAACCACTAAACACTCTAGCCTCTGCACCTGGGTCAACTGATACGGTGCTTTGGCCGTGACAATGCTCGTAAAGATTGCCACCAG
GGCGAAGGAAGAAATGACTTCGCCTCCCGTATCTGGAAAGGTGTACAT
>4 ECOL thiM TPP
ACGACTCGGGGTGCCCTTCTGCGTGAAGGCTGAGAAATACCCGTATCACCTGATCTGGATAATGCCAGCGTAGGGAAGTCACGGAC
CACCAGGTCATTGCTTCTTCACGTTATGGCAGGAGCAAACTATGCAAGTCGACCTGCTGGGTTCA
..((((..(((((...(((.((......)).)))...)))))...((.((((..((((......))))..))))))))))..((((
(..((((((.((((((((((((.....))).)))))))))(((.....))).))))))...)))))).
.(((((..(((((...(((.((......)).)))...))))).......((......))....(((((((((.((((((.((..(((
(....))))..)))))))))).)))))..)))))...(((.....))).))))))((((...))))...
>5 add Adenine
TTCGGCGATCAACGCTTCATATAATCCTAATGATATGGTTTGGGAGTTTCTACCAAGAGCCTTAAACTCTTGATTATGAAGTCTGT
CGCTTTATCCGAAATTTTATAAAGAGAAGACTCATGAATTACTTTGACCTGCCGAAGATCG
(((((((.((((............((((((.........))))))........((((((.......)))))).((((((.((((.(
(.((((((..........)))))).)))))))))))))......))))..)))))))).....
(((((((.((((.((((((((...((((((.........))))))........((((((.......))))))..))))))))((.(
(.((((((..........)))))).))))...............))))..)))))))).....
>6 xpt Guanine11
AATATAATAGGAACACTCATATAATCGCGTGGATATGGCACGCAAGTTTCTACCGGGCACCGTAAATGTCCGACTATGGGTGAGCA
ATGGAACCGCACGTGTACGGTTTTTTGTGATATCAGCATTGCTTGCTCTTTATTTGAGCGGGCAATGCTTTTTTTA
..............((((((((.....(((((........)))))..........((((((.......))))))..))))))))).(((
(.((((((((........)))))))))))......((((((((((((((.......)))))))))))))).......
..........(..(.((((.((((..(((((.......)))))..........((((((.......))))))......((((((((
((((((((........)))))).............))))))))))))).)))).))))).)..)............
>7 ydhL pbuE Adenine
TCACTTGTATAACCTCAATAATATGGTTTGAGGGTGTCTACCAGGAACCGTAAAATCCTGATTACAAAATTTGTTTATGACATTTT
TTGTAATCAGGATTTTTTTTA
......................((((((((..((........))..))))))))(((((((((((((((((..((((...))))..))
)))))))))))))))......
....(((((...((((((.........))))))........(((((.........)))))..)))))...(((.((((((......
)))))).))).........
```

```
>8 GEMM CDA
CGAAAGGGCAAACCGGTACGAAAGTCCGGGACGCAAAGCTACGGGTCCTTAAGTTCCATGGGGAATAGGACGGCTGAGCCGCTGGG
GTTATTACTTTCGCGGAGCCGCCCTATGGGGCGGTTTTTAT
>9 FNUC PREQ1
AGTAGATGTGCTAGCAAAACCATCTTTAAAAAACTAGACTTGGGGTGCAAGTCCCCTTTTTTATTGCTT
>10 VEGFA
AGACACACCCACCCACATACATACATTTATATATATATATATTATATATATATAAAAATAAATATCTCTATTTTATATATATATAAAA
TATATATATTCTTTTTTTAAATTAACAGTGCTAATGTTA
.((((....(((.............(((((((....(((.....)))..))))))).................(((((((((...)
))))))))...................)))....)))).
......................(((((..((((((((((((((.(((((((((((((.............))))))))))))).))
)))))))))..................)))..)))))..
>11 thiC TPP A.thaliana
AAAAAAAACTGCACACTCCCTGCGCAGGCATTACCCTGTTCAGGTTCAAAGGGACTTTCTCAGCCTGGTCCCGGATAGCCCGCCTT
TTCGCAGGCTATCCTGGTTCAAGCACCCCTGGTGCTTTTGCATGTCAATTCACCAACCAAATCACGCAAAAGCACCAGGGGTGCTT
GAACCAGGATAGCCTGCGAAAAGGCGGGCTATCCGGGACCAGGCTGAGAAAGTCCCTTTGAACCTGAACAGGGTAATGCCTGCGCA
GGGAGTGTGCAGTTTTTTTTTTTTTCCTGTAGCTTTCTAAAGGAGAAGAAGCTACTGTTGCCGCTCGAGTCTCGTTCCACGGTTTTC
AACAGTTAGTTTCTTATGAGCTAAGAGATTCAGC
#Selected Final
GTGATTTGGTTGGTGAATTGACATGCAAAAGCACCAGGGGTGCTTGAACCAGGATAGCCTGCGAAAAGGCGGGCTATCCGGGACCA
GGCTGAGAAAGTCCCTTTGAACCTGAACAGGGTAATGCCTGCGCAGGGAGTGTGCAGTTTTTTTT
..............................((((((((((..((((..((.(((((((((((......))))))))))).))..))
)).........)))))).....((((..((((......))))..))))..).))))..........
>12 lysC Lysine
CAACGAGATAGCCCTCCAAGAAAATGATTTCTTGACAGCCTTACATTTATTCAATGCACGGCCAGCAAATAATACCGATGGGGTTT
TATTTGCTTCGGCGACGCTCCCCTTTCAGCCTTTTTCACAGAATCCATCTTTCTCCAAAGGCATACTCTTGAAGTTCGCACCTCTA
TCTTCACC
#reversed and complemented with more nucleotides
GGTGAAGATAGAGGTGCGAACTTCAAGAGTATGCCTTTGGAGAAAGATGGATTCTGTGAAAAAGGCTGAAAGGGGAGCGTCGCCGA
AGCAAATAAAACCCCATCGGTATTATTTGCTGGCCGTGCATTGAATAAATGTAAGGCTGTCAAGAAATCATTTTCTTGGAGGGCTA
TCTCGTTGTTCATAATCATTTATGATGATTAATTGATAAGCAATGAGAGTATTCCTCTCATTGCTTTTTTT
.....((((((....((((.((((........((((((......(((.....))).....)))))).......))))..))))...
(((((((((.(((.....))).)))))))))((((.((((((.....)))))).)))).((((((((....))))))))....)))
))).................................. ((((((((((((.....)))))))))))).....
........(((....((((.((((........((((((......(((.....))).....)))))).......))))..))))...
(((((((((.(((.....))).)))))))))((((.((((((.....)))))).)))).((((((((....))))))))....)))
((((((((((............................))))))))))......................
>13 BSUBT yitI SAM
AAAAAAGTCCTCTTAAGAAGAAGACTTTGTCAGTGATTTTGTCTCTTCTTATCTTCCAAGCTGTTCGAGCTTGCTGGATTTAGCAC
CTTGGTCATGGCTGATCGCCATTACACCGGTTGCTGAAGCTTCGTCGGGCCAGTCCCTCTGCTTCTCTTGATAAGAACGGATAT
#reverse complemented
ATATCCGTTCTTATCAAGAGAAGCAGAGGGACTGGCCCGACGAAGCTTCAGCAACCGGTGTAATGGCGATCAGCCATGACCAAGGT
GCTAAATCCAGCAAGCTCGAACAGCTTGGAAGATAAGAAGAGACAAAATCACTGACAAAGTCTTCTTCTTAAGAGGACTTTTTT
.......((((((((.....((((...(((.....)))......)))).(((.((((((...(((((.....))))).)))..)))
)))........((((((.....))))))...))))))))....................((((((((...))))))))......
.............((.....((((...(((.....)))......)))).(((.((((((...(((((.....))))).)))..)))
)))........((((((.....))))))...))(((((((((((...............))).)))))))).............
>14 folT THF
TATGAAGGCAGAGTAGGTGTTATGCGTTAAGTGTTAGGGGATGGGAAGTTGCTCCTAAACGAAGAACTCATTTCGCGATATAACAC
CGCGTCCACTGTCACAGTCGAGGGAAGCTACTTTTTCGATACATTAATTTCTCTTACATGTGGCGCTATGTCTAA
......(((((....((((((((((.....((.(((((((((.....))..)))))))))...(((.....)))...)))))))))
).......))))).............................................................
>15 metA SAH
AAAAACGAACGGCGCCGCAAACCGGGGCAAGGAAAACGCGGGTTTGCGAGCGCCGTTGATCATTGCCGAGCCTGGCCCCCGAATTA
AAGGGTCGCAGCGCTCCTCGGCAGA
#reversed and complemented
TCTGCCGAGGAGCGCTGCGACCCTTTAATTCGGGGGCCAGGCTCGGCAATGATCAACGGCGCTCGCAAACCCGCGTTTTCCTTGCC
CCGGTTTGCGGCGCCGTTCGTTTTT
```

```
..((((((.[[[[[[[[...(((((......)))))......))))))........]]]]]]]]......................
.........................
>16 B. cereus crc Flouride
UAGGCGAUGGAGUUCGCCAUAAACGCUGCUUAGCUAAUGACUCCUACCAGUAUCACUACUGGUAGGAGUCUAUUUUUUU
>17 ydaO ATP
GAAAACAAATCGCTTAATCTGAAATCAGAGCGGGGGACCCAATAGAACGGCTTTTTGCCGTTGGGGTGAATCCTTTTTAGGTAGGG
CTAACTCTCATATGCCCGAATCCGTCAGCTAACCTCGTAAGCGTTCGTGAGAGGAGATGAATGAAACCTGTGTTCGATGTTATGGC
ACAGGGGCATCCGTTTGCCTCTGTGTTTTTT
>18 btuB B12
GCCGGTCCTGTGAGTTAATAGGGAATCCAGTGCGAATCTGGAGCTGACGCGCAGCGGTAAGGAAAGGTGCGATGATTGCGTTATGC
GGACACTGCCATTCGGTGGGAAGTCATCATCTCTTAGTATCTTAGATACCCCTCCAAGCCCGAAGACCTGCCGGCCAACGTCGCAT
CTGGTTCTCATCATCGCGTAATATTGATGAAACCTGCGGCATCCTTCTTCTATTGTGGATGCTTTACAATGA
>19 metA SAMII
GGCCGAAAATGGAAAGCCGAAACCCCGATGAAATCAGCCTATATTGATTTTCGCCGGCTTCGGGATTATGAAACGCCTCAGTGGTG
ATTTGCCGACCGGCTTGCAGCCACTTTAAAGAAGTCGCTAAAGGGTCGAGGAAAAGGGCAATTTCCTGGGACCGGCCGCGATTTCG
CTGCCGGTTTTTTT
>20 PH alx
TGCAAAGGGGAGTAACTTCATTGCCGGTCGATCGTCATTACGATGTGTGAAAAAACACATCCGGTCACCGGGCAACCCGAAAGGAA
TACGCAGACGTATTCCTTTTTTGTTGTAAGTGAGACCTTGCCGGAAGGCGAGGTCTATGCATAAAAAGCAGCGGCTGACGTCTTCC
GACGTTGGCCGTTTTTTTATGTGTAAGGAACTTCTATGAA

#Negative control [From Rfam database1]
>MS2 RNA (Groenveld et. al. RNA 1995 1:79-88.)
GGGTGGGACCCCTTTCGGGGTCCTGCTCAACTTCCTGTCGAGCTAATGCCATTTTTAATGTCTTTAGCGAGACGCTACCATGCTAT
CGCTGTAGGTAGCCGGAATTCCATTCCTAGGAGGTTTGACCTGTGCGA
>MDV 1 RNA (Kramer et. al. Nucleic Acids Research 1981 9:5109-5124.)
GGGGAACCCCCCTTCGGGGGTCACCTCGCGCAGCGGGCTGCGCGAAGGGGCCACGCTGCGAAGCAGCGTGGCGGTTCTCGTGGTTA
CCGAAACGCACGAAG
>5S rRNA
>ENAjHQ270648jHQ270648.1 Aegilops crassa var. glumiaristata clone CG1285- 4 5S
ribosomal RNA gene, complete sequence.
1:371-489
AATGCGATCATACCAGCACTAAAGCACCGGATCCCATCAGAACTCCGAAGTTAAGCGTGCTTGGGCGAGAGTAGTACTAGGATGGG
TGACCTCCTGGGAAGTCCTCGTGTTGCATTCC
>tRNA
>Escherichia coli CFT073 tRNA jEMBL:AE016765/complement(78960-79052)jWelch R.A., et
al. (2003) Proc. Natl. Acad. Sci.
U.S.A. 99(26):17020-17024.
GGTGAGGTGGCCGAGAGGCTGAAGGCGCTCCCCTGCTAAGGGAGTATGCGGTCAAAAGCTGCATCCGGGGTTCGAATCCCCGCCTC
ACCGCCA
>IRES Pesti
>ENAjU45478jU45478.1 Hog cholera virus strain Glentorf, complete genome. 88 -361
AGTAGGACTAGCAAACGGAGGGACTAGCCATAGTGGCGAGCTCCCTGGGTGGTCTAAGTCCTGAGTACAGGACAGTCGTCAGTAGT
TCGACGTGAGCAGAAGCCCACCTCGAGATGCTACGTGGACGAGGGCATGCCCAAGACACACCTTAACCCTAGCGGGGGTCGCTAGG
GTGAAATCACACCACGTGATGGGAGTACGACCTGATAGGGTGCTGCAGAGGCCCACTATTAGGCTAGTATAAAAATCTCTGCTGTA
CATGGCACATGGAGT
>Hammerhead ribozyme
>ENAjDQ680728jDQ680728.1 Peach latent mosaic viroid variant 107.1, complete genome. :
Location: 283-336
GAAGAGTCTGTGCTTAGCACACTGATGAGTCTCTGAAATGAGACGAAACTCTTG
>Intron GPI
>ENAjAY101137jAY101137.1 Porana volubilis tRNA-Leu (trnL) gene, partial sequence;
trnL-trnF intergenic spacer, complete
sequence; and tRNA-Phe (trnF) gene, partial sequence; chloroplast genes for
chloroplast products. Location: 4-325 RF00028
CTACGGACTTAATTGGATTGAGCCTTGGTATGGAAACTTACTAAGTGATAACTTTCAAATTCAGAGAAACCCTGGAATTAATAAAA
TGGGCAATCCTGAGCCAAATCCTGTTTTCTGAAAACAAACAAAAGTTCAGAAAAAAGGATAGGTGCAGAGACTCAATGGAAGCTGT
```

TCTAACAAATGGAGTTGACTGTATTGAAGAAAGAATTGAATATTCATTGATTAAATCATTCACTCCATAGTATAGTCTGATAGATC
TTTTGACGAACTGATTAATCGGACGAGAATAAAGATAGAGTCCTGTTCTACATGTCAATACC
>Ribosomial S15
>gbjCP000880.1j:4262517-4262631 Salmonella enterica subsp. arizonae serovar 62:z4,z23:--, complete genome
TGGGATCGCTGAATTAGAGATCGGCGTCCTTTCATTCTTAATAATTTGGAGTTTTAAAATGTCTCTAAGTACTGAAGCTACAGCTA
AAATCGTTTCTGAGTTTGGTCGTGATGCA
>U1 RNA
>ENAjAADB02000206jAADB02000206.1 Homo sapiens chromosome 1 CRA 219000002706426, whole genome shotgun sequence.: Location:
2971-3132
AAAGAGTGAGGCGTATGAGGCTGTGTCGGGGCAGAGGCCCAAGATCTCATACTTACCTGGCAGGGGAGATACCATGATCACGAAGG
TGGTTTTCCCAGGGCGAGGCTTATCCATTGCACTCCGGATGTGCTGACCCCTGCGATTTCCCCAAATGTGGGAAA
>Hammerhead type II Marine Metagenome
1 http://rfam.xfam.org/
17
GCGUGUCGGCCACGGCCCCUUCUGGACCUCGUCCGUGGCCCUGACGAGUAGGGUCCAGAGGGGACGAAACACGC

>IMES-2
>gbjAACY021487954.1 j:462-602 Marine metagenome 1093018652999, whole genome shotgun sequence
TTATGAATTAAGAGGCAACTCTTAACTGACCATCTGGGGAAAAACCGAGAGGTTCAAGCCCAGAGGGCAGAAAACTCTACAGAGTA
GCGCTAAAATTGTAGAGTGGAAGGCATGGCGGTAGCGCATACAACGACGTGCCAA
>23S-methyl
>gbjAAJQ01000051.1j:10446-10544 Streptococcus agalactiae CJB111 s agalactiae cjb111 41, whole genome shotgun sequence
GATTTGTTAGTTTAAATCAAACCTGTTATGATTTAAGCTAACAAACCACCATAAACCACATTGTTTGCTGAGATTGACTCCGAGCA
GTGTGGTTTTTTT
>mir-1937
>gij20772799:3858-3978 Mus musculus whole genome shotgun assembly contig 198984, whole genome shotgun sequence
GTCTCGGTCTCTTGGGTGGTCGCGTCAATCCCGGACGAGCCCCCAAATGAAAGATCCCCAAAGGGAGACCCTCACTCAAGCCTCGG
GATGTGGCGCGGACCCAAGAGAGACGCGGAGACCG
>RNaseP nuc
>gbjAANG01621100.1j:3337-3671 Felis catus breed Abyssinian cont1.621099, whole genome shotgun sequence
GGCGGAGGGAAGCTCATCGGTGGGGCTACGCGCTGAGTGCCTTAAGTCACTCTACCCCATGTCCCCTGGGAAGGTCTGAGACTAGG
GCCAAAAGCGGCCCTAACAGGGTCCTCCCTGAGTTGCGGGGAGGTGAGTTCCCAGAGAACGGGGCTCCGCGCGAGGGCAGACTGGG
CAGGAGATGCCATGGACCCCGCCCTCGAGGAGGGGCCTGGCGGATGCCTCCTTAGCCGGAGCTTGGAAAAGACTCACGGCCAGCGA
AGTGAGTTCAATGGCTGAGGTGAGGTACCCCCCAACGGGGGGGCCTCATAACCCAATTCAGACTACTCTCCCCCGTC
>t44
>gij330859585:12225-12339 Yersinia enterocolitica W22703 biovar 2, serovar O:9, contig 7180000001189
ACTCATATGTGTAAATAACACACACGTGTCGACACATACGCCGGGGTGCCCTAAAGTTTTTGATTATCATCAATCGCTTATGGGGT
CGGCGTTATGGGACACGTGGAGGCATAAC
>Phe leader
>gbjABWM02000032.1j:6775-6899 Enterobacter cancerogenus ATCC 35316 E cancerogenus-1.0.1 Cont2.3, whole genome shotgun sequence
TATGAAACATACGCCGTTTTTCTTCGCATTCTTTTTTACCTTCCCCTGATTGGGAGGCGTTTCGTCGTGTGATAAAGAATGCGAAG
ACGAACAACAAGGCCTCCCTACCGGGAGGCCTTTTTTAT
>U6atac
>gbjAAYZ01599293.1j:336-461 Ochotona princeps cont2.599292, whole genome shotgun sequence
GTGTTGTATGAAAGGAGAGAAGGTTAGCACTCCCCTTGACAAGGATGGAAGAGGCCCTCGGGCCTGACAACACACATACGGTTAAG
GCATTGCCACCTACTTCGTGGCATCTAACCACTGTTTTTT
>RsaD
>gbjACRM01000027.1j:12785-12974 Staphylococcus hominis subsp. hominis C80 cont1.27, whole genome shotgun sequence

GTTTGCTATACTATGTATAACTTCTTAATTACAAGAAGTTATCTCCTTTGTGTTGTTTATAGTAACAAAACAAATTTTGCTCGAAG
TACTGTGACAGTACCTAGTCCTTACCGTTACAAGTACATTAATATTCATTTCCCATAAAAACCAAGTATATAAAGTGTCTCCTCAC
TTTGCATGCTTGGTTTTT
>S pombe snR93
>gij61161937jembjAJ632011.1jSchizosaccharomyces pombe snR93 snoRNA gene
TTGCACTTTTTAACAAAATTACCCGTGAGATTAATTGCGGTTGAACTTGTTTTTCGTGCTAGACTGATGATTCGCCCTTATACATA
TTGGCTAACTTTCGGCTAATCTTCACCGAATTTAGTCAAGAATTGGGTGGTACAAAT
>rivX
>gbjAAFV01000140.1j:1455-1702 Streptococcus pyogenes M49 591 sp001.239.1368, whole genome shotgun sequence
AGCCTTGCACCAGAACCACTACCAAAAACGTTTAGAAGAACTTTGGAGAGTTCCTTCTTAATTTTTTGACAGTCCCTCCTAGATAC
TTTCACAGTCTAGGGGGGTCTTTAAGGTTGTCACTTGCAACCTTGATAAGCTCTCTTTTTAACCATTGCTATTCGCCTAGAAACAG
CACTGGAACTGTTTTAGCGGTTCAACTCCTCTTGTAGGATCTTCTTCCCTTTTTTGAAGGTCTTATCTGAAATTTG
